# Evaluation of associations between asthma exacerbations and distance to roadways using geocoded electronic health records data

**DOI:** 10.1186/s12889-020-09731-0

**Published:** 2020-10-29

**Authors:** Jingyi He, Mohsen Ghiasi Ghorveh, Jillian H. Hurst, Monica Tang, Brooke Alhanti, Jason E. Lang, Benjamin A. Goldstein

**Affiliations:** 1 Department of Biostatistics & Bioinformatics, Duke University, 2424 Erwin Road, Durham, NC 27705 USA; 2 Duke Clinical Research Institute, Durham, NC USA; 3 Children’s Health & Discovery Initiative, Duke University, Durham, NC USA; 4 Department of Pediatrics, Duke University, Durham, USA; 5 Department of Medicine, University California, San Francisco, USA

**Keywords:** Air pollution, Asthma, Electronic health records, Survival analysis

## Abstract

**Background:**

Asthma exacerbations in children often require medications, urgent care, and hospitalization. Multiple environmental triggers have been associated with asthma exacerbations, including particulate matter 2.5 (PM2.5) and ozone, which are primarily generated by motor vehicle exhaust. There is mixed evidence as to whether proximity to highways increases risk of asthma exacerbations.

**Methods:**

To evaluate the impact of highway proximity, we assessed the association between asthma exacerbations and the distance of child’s primary residence to two types of roadways in Durham County, North Carolina, accounting for other patient-level factors. We abstracted data from the Duke University Health System electronic health record (EHR), identifying 6208 children with asthma between 2014 and 2019. We geocoded each child’s distance to roadways (both 35 MPH+ and 55 MPH+). We classified asthma exacerbation severity into four tiers and fitted a recurrent event survival model to account for multiple exacerbations.

**Results:**

There was a no observed effect of residential distance from 55+ MPH highway (Hazard Ratio: 0.98 (95% confidence interval: 0.94, 1.01)) and distance to 35+ MPH roadway (Hazard Ratio: 0.98 (95% confidence interval: 0.83, 1.15)) and any asthma exacerbation. Even those children living closest to highways (less 0.25 miles) had no increased risk of exacerbation. These results were consistent across different demographic strata.

**Conclusions:**

While the results were non-significant, the characteristics of the study sample – namely farther distance to roadways and generally good ambient environmental pollution may contribute to the lack of effect. Compared to previous studies, which often relied on self-reported measures, we were able to obtain a more objective assessment of outcomes. Overall, this work highlights the opportunity to use EHR data to study environmental impacts on disease.

**Supplementary Information:**

**Supplementary information** accompanies this paper at 10.1186/s12889-020-09731-0.

## Background

Asthma is one of the most common chronic respiratory diseases, affecting more than 25 million Americans, including 9 % of children in the United States [1, 2]. Asthma exacerbations are acute episodes of worsening shortness of breath, wheezing, cough, and chest tightness that cause the majority of healthcare utilization and morbidity associated with asthma [3]. Over three million children suffer from at least one asthma exacerbation per year, with more than 10% of exacerbations requiring hospitalization [4]. Various clinical and environmental risk factors for asthma exacerbations have been identified. Previous studies have shown that outdoor air pollution, including fine particulate matter and gaseous pollutants from traffic and power generation can increase symptoms in children who have already been diagnosed with asthma [5–9].

Specifically, particulate matter 2.5 (PM2.5) and ozone (O_3_), which form from motor vehicle exhaust, are well studied environmental triggers of asthma symptoms [10–12]. Areas close to major roadways can have high PM2.5 and CO levels [13, 14], and there is a growing body of evidence suggesting that proximity to car traffic sources can negatively affect asthma severity [15–25]. Importantly, the results of these studies are somewhat mixed, with a few studies either finding no association or weak associations between asthma exacerbations and distance to roadways [6, 26–28]. Some of the inconsistency in the results can be attributed to heterogeneity in study design, outcomes, cohort definition, and exposure assessments [28]. In particular, the majority of studies used self-reported questionnaires to collect exposure and outcomes data, which can result in potential misclassification and recall bias [26]. Thus, further studies are needed to evaluate the impact of distance to roadways on pediatric asthma exacerbations.

In this study, we sought to assess the association between the distance to two types of roadways (35+ MPH and 55+ MPH) and rates of pediatric asthma exacerbations in a medium sized eastern US city. To account for potential biases in the self-reporting of outcomes, we relied on data abstracted from our institution’s electronic health record (EHR) system to objectively obtain information on asthma exacerbations.

## Methods

### Data sources

All data were extracted from the Duke University Health System Electronic Health Record (EHR) system. Duke University Health System consists of 3 hospitals – 1 tertiary care and 2 community-based – and a network of primary care and specialty clinics. As the primary provider in Durham County, North Carolina, it is estimated that 80% of Durham County residents receive their care through Duke University Health System [29]. For the purposes of this study we abstracted analytic data from our data warehouse covering the years January 1, 2014 to December 31, 2019.

### Study population

We identified children (age 5–18), living in Durham County with asthma. Children had to be at least 5 years old to rule out unclear respiratory related diagnoses in younger children. To identify children with asthma we applied two definitions. Our first definition was encounter-based. A child had to have: 1) two outpatient encounters or one inpatient encounter with an asthma diagnosis (see Appendix Table 1 for ICD9/10 codes) and 2) a prescription for an asthma medication (see Appendix Table 2). Our second definition was problem list-based. Problem lists are an EHR feature that serves as a comprehensive list of patient diagnoses that is intended to serve as a snapshot of the patient’s health status. To be included under the problem list-based definition, a child had to have 1) asthma on their problem lists and 2) a prescription for an asthma medication. The positive predictive value of this computable phenotype is 97% [30].

In order to account for differential follow-up times, person-time was calculated from time of positive asthma identification until censoring. Censoring was based on aging out of the cohort (> = 18), an indicated address outside of Durham County, or at the last known encounter.

### Primary exposure

The primary exposure in our study was the residential distances to two types of roadways: roads with U.S. Census feature Class Code A1 (55+ MPH with limited access only accessible via ramps) and A2 (35+ MPH-primary road without limited access) [31–33], with these speeds corresponding to highways and major roadways, respectively [15, 16, 18, 24]. We abstracted the address information of each individual in our cohort from our EHR system and geocoded them using ArcGIS (version 10.5; ESRI Inc., Redlands, CA). The accuracy of all address information was manually checked with Google Maps to verify the existence of residences at each address. Addresses were treated as a time-varying exposure based on when a child moved to a new address, as indicated in the EHR. Map figures were made with ArcGIS software.

**Fig. 1:**
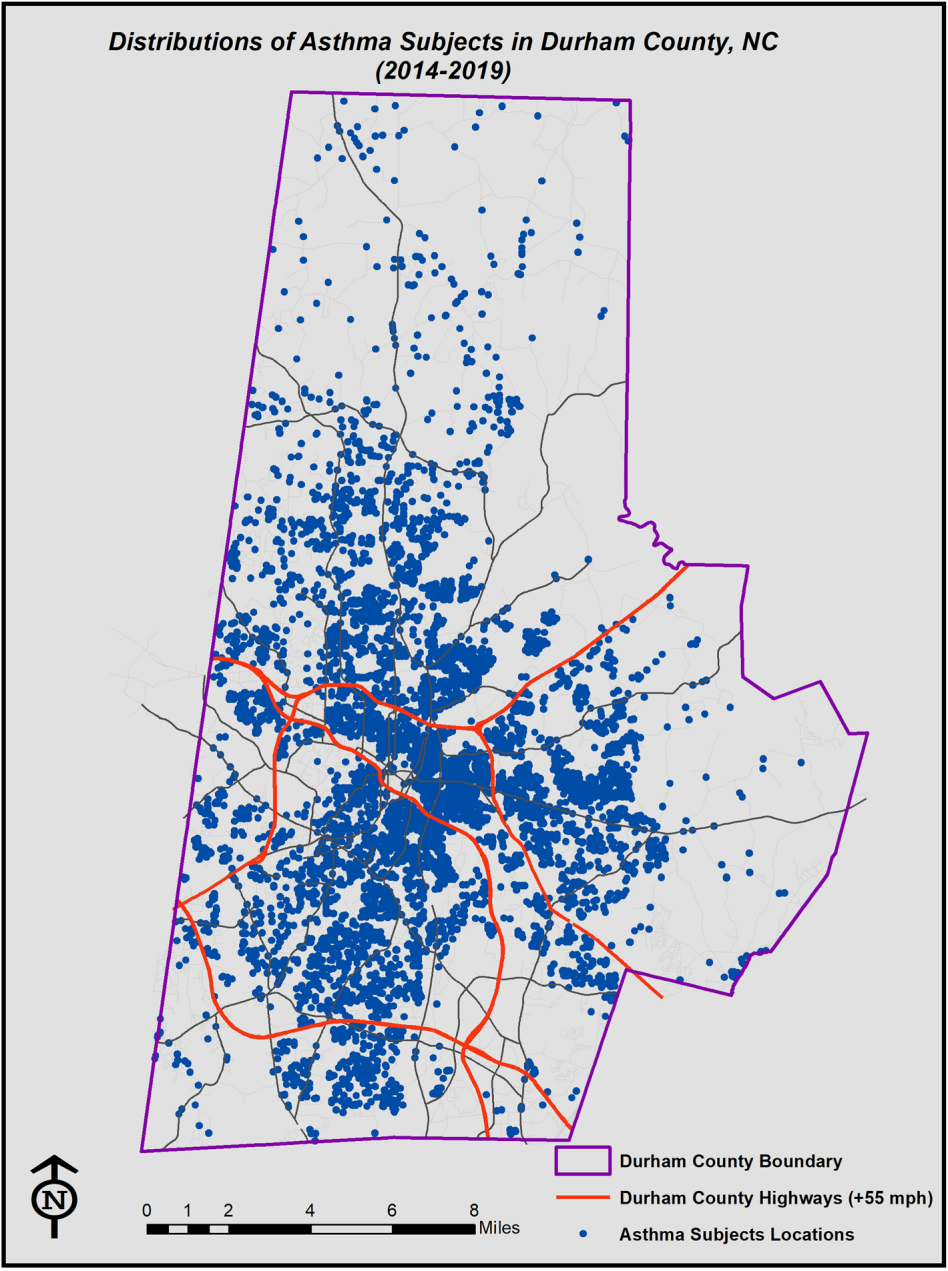
Living location of children in our cohort and proximity to major roadways in Durham County. These blue dots represent the living location of every children with asthma in our cohort, and the red lines are the 55+ MPH highways in Durham. The edge of the county is the rural area. This map was generated with ArcGIS software

**Fig. 2:**
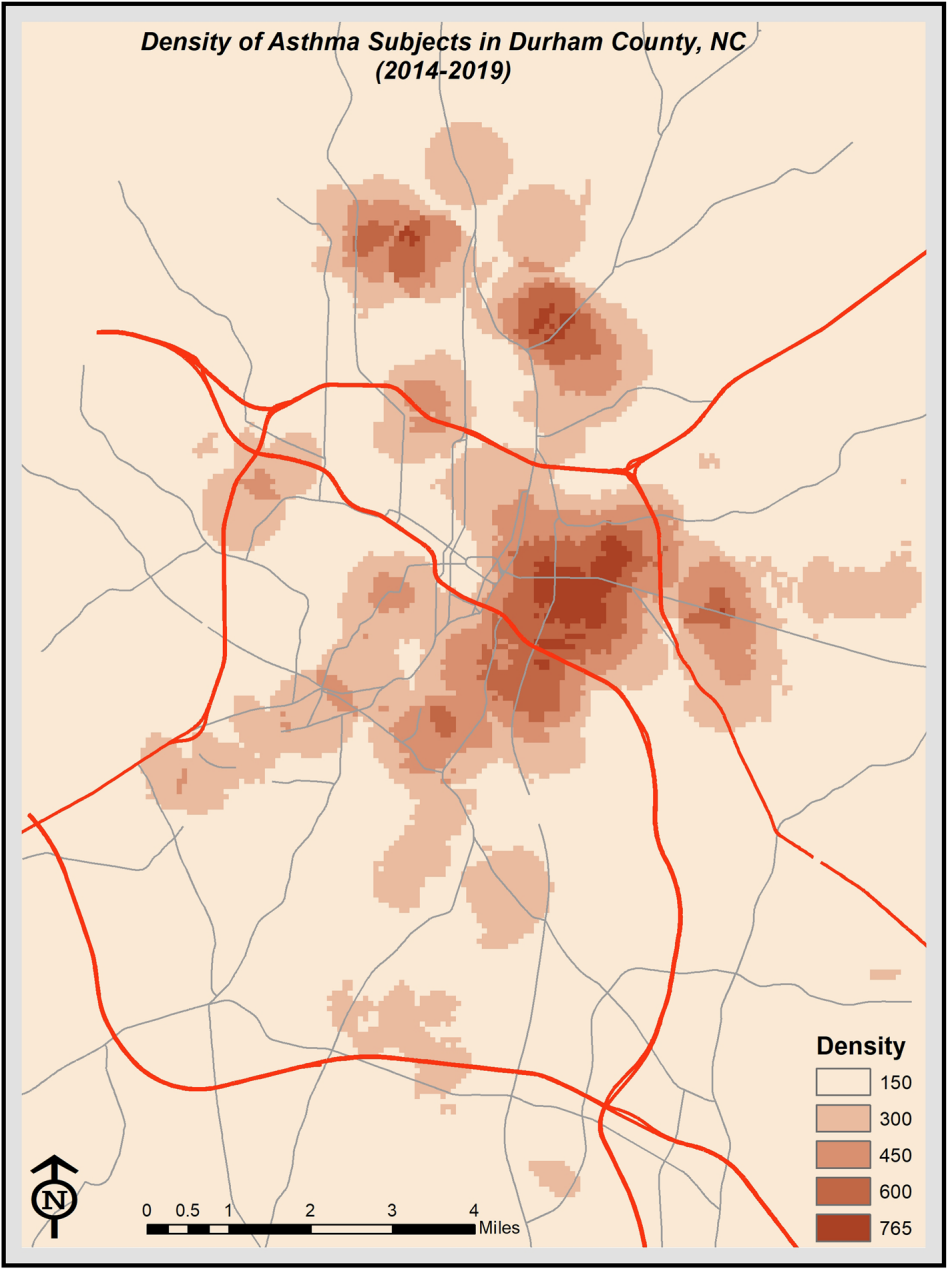
Demonstration of radial density measures of 1.0 mile: we calculated the length of each type of highways within in a 1-mile buffer around each geocoded address location. This map was generated with ArcGIS software

Straight-line distance to roadways, which was calculated using ArcGIS, was used as our primary exposure (See Fig. 1). Durham County has three major roadways that intersect in central Durham (Fig. 1). For children who live within this triangular intersection, straight-line distance might not accurately capture exposure. We therefore also constructed radial density measures of 1.0 mile in order to evaluate exposures associated with proximity to more than one roadway (Fig. 2).

### Outcome of interest

The primary outcome of interest was an asthma-related exacerbation, which was defined as any encounter with an asthma-related ICD9 or − 10 code and a prescription for a systemic steroid (see Appendix Table 1). We further categorized exacerbations into four different outcome tiers based on severity (listed in decreasing severity): 1) inpatient encounters lasting more than 24 h, 2) emergency department and hospital encounters lasting less than 24 h, 3) urgent care visits, and 4) outpatient (including telephone-based) encounters.

### Covariates

We abstracted additional clinical and socio-demographic information on each child from the EHR, including sex, age, race, insurance type (public, private, self-pay), neighborhood socio-economic status (nSES), comorbidities (atopy, obesity), medication categories (only rescue, only inhaled corticosteroids or only Leukotriene receptor antagonist, or other controller medications), and number of overall encounters. Covariates were treated as time-varying and abstracted accordingly. We considered any prescription order in the past 365 days as current. To calculate nSES, we identified each child’s census tract and linked data from the American Community Survey to calculate the Agency for Healthcare Research and Quality (AHRQ) neighborhood deprivation index [34], generating a score between 0 and 100, with higher scores indicative of greater deprivation.

### Air quality exposure

To further characterize potential exposures, we abstracted publicly available data from Environmental Protection Agency sensors on daily PM2.5 [35]. We note that Durham County only has one sensor, providing a general approximation of daily PM2.5 exposure.

### Statistical analysis

We constructed a time-varying dataset in counting-process format, generating an encounter row each time a child moved (i.e., changed primary exposure) or had an asthma exacerbation. All time-varying covariates (e.g., medications) were calculated based on these encounter points. We categorized patients based on their baseline distance to roadway, compared clinical and demographic differences among these groups, and calculated the number of exacerbations per patient-year.

To assess the relationship between distance to roadway and asthma exacerbation rates we performed a survival time-to-event analysis, treating distance as a time-varying exposure. We fit separate models for straight-line distance and roadway radial density. In our cohort, about 25% of children had more than one asthma exacerbation over the study period. To account for multiple exacerbations, we conducted a recurrent events survival analysis using Andersen-Gill models [36]. As a sensitivity analysis, we used the Prentice, Williams and Peterson model and frailty model (random effects approach) as alternative recurrent events models [37]. Four different models were fit: 1) unadjusted; 2) adjusted for nSES; 3) adjusted for sex, age, race, and insurance type; and 4) adjusted for all the other covariates specified above. Additionally, we assessed the linearity of the effect of distance to roadways and roadway radial density on asthma exacerbation outcomes. We categorized our distance to roadways variables into quartiles and refitted the models. We also stratified on sex, race, and assessed time to outcome specific tiers.

All the statistical analyses were perform using R version 3.6.0.

## Results

Our analytic cohort consisted of 6395 children with asthma, 187 of whom had invalid addresses and were excluded. Among the remaining 6208 children with asthma, we identified 6511 unique addresses and 3739 asthma exacerbations (2 exacerbations per 10 patient years). Around half of the unique addresses represented in the cohort were within 1 mile of a 55 MPH+ roadway among the unique addresses. Baseline characteristics are shown in Table 1. We first categorized patients based on their baseline initial straight-line distance to roadway: less than 0.25 mile, 0.25–0.5 mile, 0.5–1 mile, and greater than 1 mile – which roughly corresponded to the 10th, 25th and 50th quartiles. The median age of the cohort was ~ 9 years old and the majority of children were African Americans (~ 60%). Lower nSES scores were associated with residential locations close to roadways. There were no observed associations between asthma-related clinical factors (i.e. atopy, obesity, medication, and number of encounters in past year) and distance to roadway.

**Table 1:** Baseline Patient Characteristics by Distance to Highway (55+) Categories

	< 0.25 mile *N* = 776	0.25–0.5 mile *N* = 988	0.5–1 mile *N* = 1545	> 1 mile *N* = 3202
Sex				
Male	441 (56.8%)	566 (57.3%)	848 (54.9%)	1803 (56.3%)
Female	335 (43.2%)	422 (42.7%)	697 (45.1%)	1399 (43.7%)
Age	8.0 (6.0–12.0)	9.0 (6.0–13.0)	9.0 (6.0–12.0)	9.0 (6.0–13.0)
Race				
Hispanic	154 (19.8%)	91 (9.2%)	221 (14.3%)	326 (10.2%)
Non-Hispanic Black	465 (59.9%)	662 (67.0%)	888 (57.5%)	1814 (56.7%)
Non-Hispanic White	90 (11.6%)	143 (14.5%)	276 (17.9%)	773 (24.1%)
Other	54 (7.0%)	74 (7.5%)	118 (7.6%)	224 (7.0%)
Unavailable/Unknown	13 (1.7%)	18 (1.8%)	42 (2.7%)	65 (2.0%)
Atopy				
No	293 (37.8%)	354 (35.8%)	628 (40.6%)	1211 (37.8%)
Yes	483 (62.2%)	634 (64.2%)	917 (59.4%)	1991 (62.2%)
Obesity				
No	561 (72.3%)	707 (71.6%)	1083 (70.1%)	2426 (75.8%)
Yes	206 (26.5%)	264 (26.7%)	446 (28.9%)	749 (23.4%)
Missing	9 (1.2%)	17 (1.7%)	16 (1.0%)	27 (0.8%)
Insurance Type				
Private	179 (23.1%)	290 (29.4%)	494 (32.0%)	1313 (41.0%)
Public	565 (72.8%)	648 (65.6%)	985 (63.8%)	1746 (54.5%)
Self-pay	29 (3.7%)	44 (4.5%)	62 (4.0%)	118 (3.7%)
Unavailable/Unknown	3 (0.4%)	6 (0.6%)	4 (0.3%)	25 (0.8%)
Medication Category				
No prescriptions	475 (61.2%)	577 (58.4%)	917 (59.4%)	1890 (59.0%)
Only ICS or only LTRA	229 (29.5%)	346 (35.0%)	510 (33.0%)	1095 (34.2%)
Combination	72 (9.3%)	65 (6.6%)	118 (7.6%)	217 (6.8%)
SES Status Category				
25–50	414 (53.4%)	663 (67.1%)	927 (60.0%)	1485 (46.4%)
50–75	260 (33.5%)	318 (32.2%)	616 (39.9%)	1717 (53.6%)
Missing	102 (13.1%)	7 (0.7%)	2 (0.1%)	0 (0.0%)
Number of Encounters in past year	2.0 (1.0–5.0)	2.0 (1.0–5.0)	2.0 (1.0–5.0)	3.0 (1.0–5.0)

*ICS* Inhaled corticosteroids, *LTRA* Leukotriene receptor antagonist, *SES* Socio-economic Status

We assessed the relationship between distance to roadway and asthma exacerbation rates. We observed negligible relationships between distance to 55+ MPH roadways and overall, inpatient, emergency department, urgent, and outpatient asthma exacerbations, both before and after adjustment (Table 2). While the results suggest some slight protective effect for living further from a roadway, most of the confidence intervals crossed 1. Examining distance to 35+ MPH roadways and asthma exacerbation rates, there was a clear lack of association, as the results were less consistent and the confidence intervals were much wider (Table 3). Results were consistent when considering alternative statistical models (Appendix Table 3).

**Table 2:** Association Between Asthma Exacerbations and Distances to 55+ MPH Highways

	Number of events	Unadjusted		+ Adjustment for SES Score		+ Adjustment for Race/Insurance/Sex/Age		+ Adjustment for Number of Encounter/ Medication/Obesity/Atopy	
		HR	95% CI	HR	95% CI	HR	95% CI	HR	95% CI
Overall	3739	0.98	0.94, 1.01	0.98	0.94, 1.02	0.99	0.95, 1.02	0.99	0.96, 1.02
Inpatient	304	1.00	0.94, 1.06	0.98	0.91, 1.05	0.98	0.90, 1.06	0.97	0.89, 1.06
ED	895	0.96	0.90, 1.02	0.97	0.91, 1.03	0.97	0.92, 1.03	0.98	0.93, 1.03
Urgent	935	0.93	0.89, 0.98	0.93	0.89, 0.97	0.94	0.89, 0.98	0.94	0.89, 0.99
Outpatient	1850	0.99	0.96, 1.02	1.00	0.97, 1.03	1.00	0.97, 1.03	0.99	0.96, 1.03

*HR* Hazard ratio 95%, *CI* 95% Confidence interval, *ED* Emergency department, *SES* Socio-economic Status

**Table 3:** Association Between Asthma Exacerbations and Distances to 35+ MPH Roadways

	Number of events	Unadjusted		+ Adjustment for SES Score		+ Adjustment for Race/Insurance/ Sex/Age		+ Adjustment for Number of Encounter/ Medication/Obesity/ Atopy	
		HR	95% CI	HR	95% CI	HR	95% CI	HR	95% CI
Overall	3739	0.98	0.83, 1.15	0.98	0.83, 1.16	1.02	0.86, 1.21	1.00	0.85, 1.18
Inpatient	304	1.25	0.89, 1.75	1.20	0.86, 1.69	1.34	0.96, 1.86	1.10	0.80, 1.52
ED	895	1.00	0.79, 1.27	1.03	0.81, 1.31	1.06	0.83, 1.35	1.07	0.83, 1.38
Urgent	935	0.88	0.70, 1.10	0.87	0.69, 1.09	0.89	0.70, 1.12	0.82	0.65, 1.04
Outpatient	1850	0.99	0.86, 1.14	1.01	0.87, 1.17	1.02	0.88, 1.18	0.99	0.85, 1.15

*HR* Hazard ratio 95%, *CI* 95% Confidence interval, *ED* Emergency department, *SES* Socio-economic Status

**Fig. 3:**
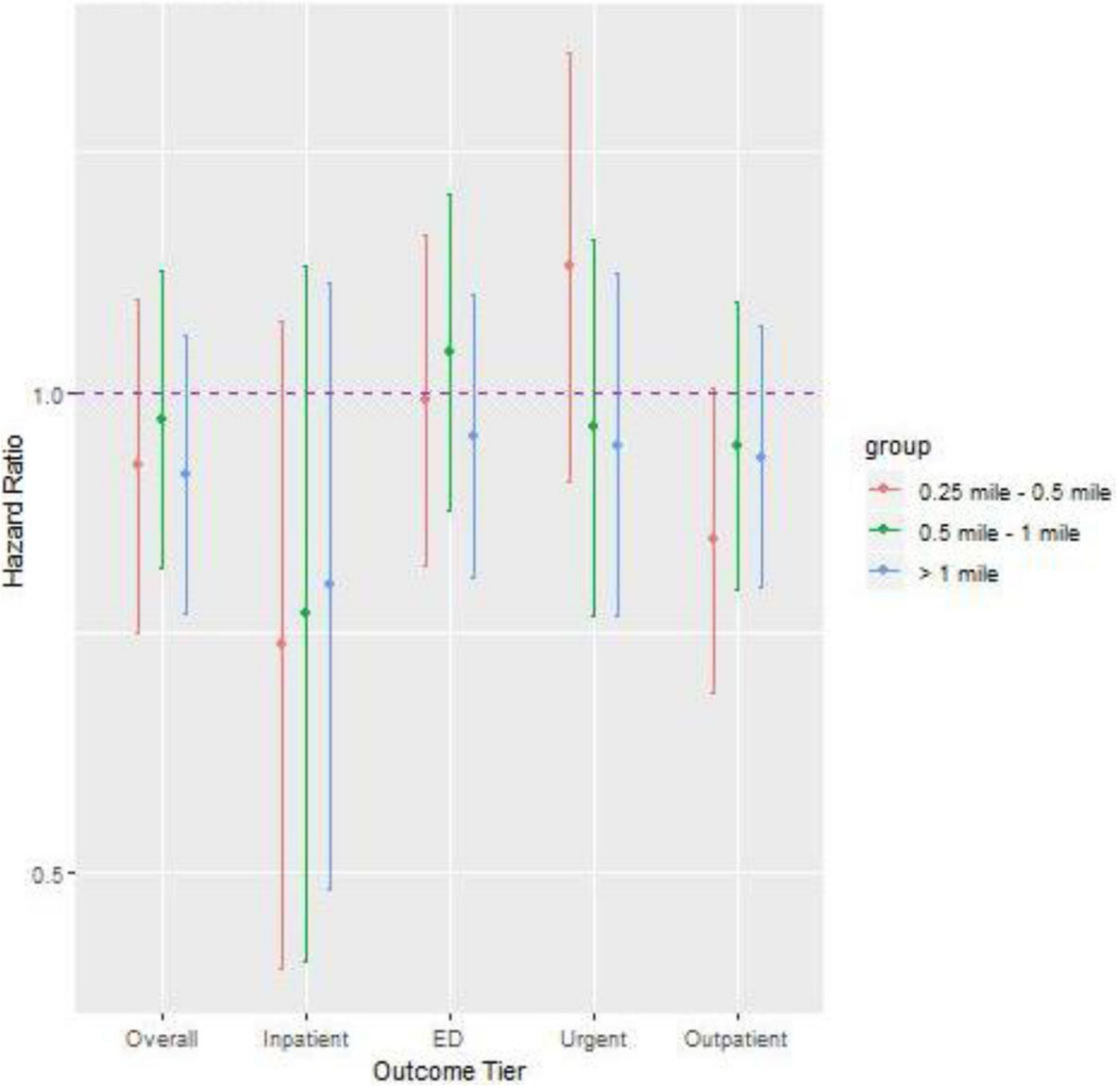
Assessment of non-linear associations between asthma exacerbation and distance to roadways among different distance groupings for different outcome tiers. The reference group here is < 0.25 mile, and there is no evidence showing children living closest to roadways had increased risk of asthma exacerbation

We further assessed the relationship between the density of roadways within census tracts and risk of exacerbation, but found minimal associations (Tables 4 & 5). Finally, we assessed whether there was a non-linear association by evaluating distance groupings of 0–0.25 miles, 0.25–0.5 miles, 0.5–1 mile, 1 mile + (Fig. 3). Overall, there was no suggestion that children living closest to roadways had increased risk of asthma exacerbation. We performed sensitivity analyses stratified on age and sex and found results consistent across strata (results not shown).

**Table 4:** Association Between Asthma Exacerbations and 1-mile Buffer Density Measurement Around 55+ MPH Highways

	Number of events	Unadjusted		+ Adjustment for SES Score		+ Adjustment for Race/Insurance/ Sex/Age		+ Adjustment for Number of Encounter/ Medication/Obesity/ Atopy	
		HR	95% CI	HR	95% CI	HR	95% CI	HR	95% CI
Overall	1854	0.98	0.93, 1.04	0.98	0.93, 1.02	0.99	0.94, 1.03	0.98	0.94, 1.02
Inpatient	168	1.01	0.92, 1.11	1.04	0.93, 1.17	1.05	0.92, 1.19	1.02	0.92, 1.13
ED	497	0.98	0.93, 1.04	0.96	0.90, 1.02	0.97	0.90, 1.03	0.98	0.92, 1.05
Urgent	497	0.98	0.9, 1.03	0.99	0.94, 1.04	0.99	0.94, 1.05	1.00	0.95, 1.06
Outpatient	824	1.05	0.98, 1.12	1.03	0.98, 1.09	1.03	0.97, 1.08	1.03	0.97, 1.08

*HR* Hazard ratio 95%, *CI* 95% Confidence interval, *ED* Emergency department, *SES* Socio-economic Status

**Table 5:** Association Between Asthma Exacerbations and 1-mile Buffer Density Measurement Around 35+ MPH Roadways

	Number of events	Unadjusted		+ Adjustment for SES Score		+ Adjustment for Race/Insurance/ Sex/Age		+ Adjustment for Number of Encounter/ Medication/Obesity/ Atopy	
		HR	95% CI	HR	95% CI	HR	95% CI	HR	95% CI
Overall	1854	1.00	0.98, 1.03	1.00	0.98, 1.03	1.00	0.98, 1.03	1.01	0.98, 1.04
Inpatient	168	1.00	0.95, 1.05	1.00	0.95, 1.05	1.01	0.96, 1.07	1.01	0.95, 1.08
ED	497	1.01	0.98, 1.05	1.01	0.98, 1.05	1.01	0.97, 1.04	1.00	0.97, 1.04
Urgent	497	0.98	0.95, 1.02	0.98	0.95, 1.02	0.98	0.95, 1.02	0.99	0.95, 1.03
Outpatient	824	1.00	0.98, 1.03	1.00	0.97, 1.03	1.00	0.97, 1.03	1.00	0.98, 1.03

*HR* Hazard ratio 95%, *CI* 95% Confidence interval, *ED* Emergency department, *SES* Socio-economic Status

**Fig. 4:**
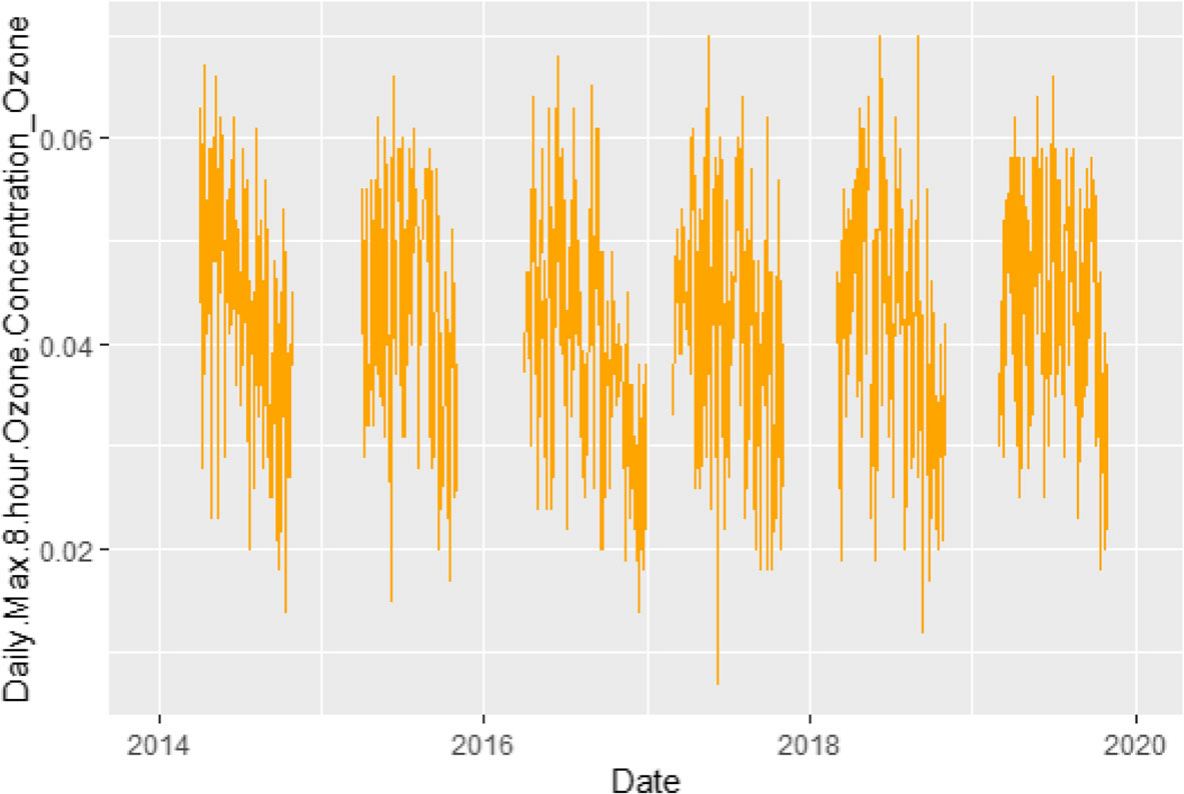
Daily 8-h maximum PM2.5 levels from 2014 to 2019. The recommended exposure limit for PM2.5 is 35 μg/m^3^. There was only one period – fall of 2017, corresponding to wild fires in the region – that exceeded this threshold

In order to further contextualize our results, we assessed daily PM2.5 levels during the study period (Fig. 4). The exposure limit for PM2.5 is 35 μg/m^3^ [38]. As shown in this plot, there was only period that exceeded this limit from 2014 to 2019 – corresponding to wildfires in the region.

## Discussion

In this study, we used EHR data to assess the association between residential distance to roadways on asthma exacerbation in children. There are a number of studies suggesting that proximity to high traffic areas, including roadways, have adverse effects on child respiratory health [39, 40], hospital admissions for asthma [41], pulmonary function [42, 43], and atopy [44], and there is some evidence that early life exposure to traffic-related pollution affects later asthma outcomes [45]. For instance, a cohort study by Deng et al. surveyed 2598 preschool children and demonstrated that maternal exposure to NO_2_ during the late pregnancy period could be associated with a higher risk of developing pediatric asthma, allergic rhinitis, and eczema [46]. Similarly, a Swedish birth cohort indicated that exposure to traffic-related air pollution during infancy could be linked to lung function decrements in children through adolescence [47, 48]. In our assessment of exacerbation rates in children with diagnosed asthma, we found a negligible relationship between distance from 55+ MPH roadway and any asthma exacerbation. Distance from 35+ MPH roads did not affect exacerbations at all.

Though our results were negative, our study has a number of strengths that differentiates it from prior work. For one, our cohort was relatively large compared with previous studies, ranging from 2 times to 15 times larger [16, 19, 24, 26, 28]. Moreover, the majority of previous studies used questionnaires to collect information [19, 24, 28]. Such questionnaires are frequently complicated and time-consuming, and, because questionnaires rely on self-reports, they can cause some potential misclassification due to recall bias for outcomes and/or exposures [26]. Instead of relying on self-report, we directly extracted patient information from the EHR system, which efficiently provides data on a large number of individuals and avoids biases found with self-reported data.

The lack of significant association between residential distance to roadway and asthma exacerbation rates could potentially be explained by factors related to air quality and the built environment of Durham. First, the air quality in Durham is generally good (Fig. 4). Other studies that reported a weak or no association between asthma and traffic-related air pollution evaluated populations living in locations with good air quality, such as Sweden, Norway, and Germany [6, 26–28]. Based on the worldwide air quality rankings for mid-2019, the air quality index for these countries were 17.45, 20.29 and 28.42 respectively [49]. In contrast, studies that reported strong associations were conducted in places such as the United Kingdom, Poland, and South Korea, whose air pollution indexes were 40.63, 52.88, and 54.80, respectively [22–24, 50]. Similar trends have been reported within the United States. A study conducted in Los Angeles reported that children and adults who suffer from asthma and live close to traffic are nearly three times more likely to visit the emergency department or be hospitalized than those who live near low traffic density [18]. In fact, cities in California, including Los Angeles, have ranked to have the worst particle pollution in the US [51].

A second reason our results may differ is that compared to other studies our cohort lived further from roadways. Multiple studies reported a threshold of 150 m (~ 0.1 mile) for finding the highest concentrations of traffic-related pollutions [17, 24]. However, in our cohort, only 283 children live within this proximity to roadways. Two previous studies in California and South Korea utilizing radial density measures similar to ours found effects only at much closer distances of 500 ft and 200 m respectively, than the 1-mile buffer we used [22, 24].

Only one other study, conducted in Minnesota, has utilized EHR data to assess the relationship between asthma exacerbation and traffic-related air pollution [52]. The authors used vehicle kilometers traveled within 250 m and 500 m of each individual’s residence, as well as the traffic density as their exposures. Traffic exposures is similar to the radial density measures in our study. Assessing both pediatric and adult patients, the authors reported that traffic exposure at the residence increases the risk of asthma exacerbations. The authors reported lower odds ratio for vehicle kilometers traveled within 500 m than the vehicle kilometers traveled within 250 m and affirmed that 500 m buffer distance does not effectively capture the traffic effect. Moreover, while air quality across Minnesota is generally within the healthy range, there are several days each year where fine particles levels can exceed to the unhealthy levels [53]. Overall this paper helps to contextualize some of the present findings highlighting that it is both close exposure to roadways that matters and that there may be air quality threshold that an environment needs to drop below.

One challenge in studying the effect of one’s distance to highway on risk of asthma exacerbation is that we are assessing the effect of chronic exposure on an acute outcome. It is likely that such chronic exposures will have a greater impact on longer term disease progression, where more acute changes in one’s environment will more directly impact one’s risk of exacerbation. Ultimately, any association between distance to roadways and asthma is going to be mediated through environmental air quality. Therefore, future work should seek to not only measure an individual’s distance to highway but the ambient air quality in their neighborhood. With newer technology in satellite imaging, it is possible to more finely map neighborhood air quality [54].

There are a few limitations in our study. While our results suggest no association between residential distance to roadway and asthma exacerbation rates, a null result is hard to prove, as statistical tests are not designed to detect them. Moreover, our results are limited to one geographic area. As we noted above, Durham County has several unique characteristics, including relatively good air quality, which may cause effect heterogeneity between studies. Additionally, highway exposure is only one form of environmental exposure that can impact risk of asthma exacerbation. Indoor air quality, seasonal factors and smoking exposure are environmental factors that we were not able to account for in this analysis. Finally, while we believe that the use of EHR data produces more objective data measurements, EHR data are prone to their own biases [55]. In particular, type of health service utilization is often a reflection of both severity of disease as well as other social factors such as SES and health seeking behavior. Of note, our analyses did not indicate confounding due to nSES.

## Conclusion

In conclusion, this study shows that proximity to roadways is not strongly associated with asthma exacerbation rates in Durham County from 2014 to 2019. Comparing these findings with reports from other locations, suggests that both close proximity and overall air quality are perhaps important modifiers of the association. Moreover, it illustrates how EHR data, combined with environmental data, can be used to assess environmental effects on population health.

## Supplementary Information

**Additional file 1.**

## Data Availability

The analysis data is not available as it contains protected health information. An anonymized data set is not available upon request.

## Abbreviations

EHR: Electronic health records
ICD: International classification of disease
GIS: Geographic information software
MPH: Miles per hour
nSES: Neighborhood socioeconomic status
PM: Particulate matter
SES: Socioeconomic status
