# Supplementary material for: Evaluation of associations between asthma exacerbations and distance to roadways using geocoded electronic health records data
Source: BMC Public Health. 2020 Oct 29;20:1626. doi: 10.1186/s12889-020-09731-0 (PMC7599107; doi:10.1186/s12889-020-09731-0)
Supplement: Supplementary file 1 — Additional file 1. [file 12889_2020_9731_MOESM1_ESM.docx]

| Appendix Table 1. Asthma related-exacerbation | | | |
| --- | --- | --- | --- |
| Diagnosis | ICD 9 | ICD 10 | Medication |
| Asthma | 493.* | J45.* | **Systemic steroids**:  o    Prednisone  o    Prednisolone  o    Dexamethasone  o    Hydrocortisone  o    Methylprednisone |
| Respiratory abnormality, unspecified | 786.00 | R06.00 |  |
| Shortness of breath  (Acute respiratory distress) | 786.05 | R06.02  R06.03 |  |
| Tachypnea | 786.06 | R06.82 |  |
| Wheezing | 786.07 | R06.2 |  |
| Other respiratory abnormalities  (Other forms of dyspnea,  Other abnormalities of breathing, Unspecified abnormalities of breathing) | 786.09 | R06.09  R06.89  R06.9 |  |
| Cough | 786.2 | R05 |  |

* Asthma codes used to define cohort, but all associated respiratory diagnoses used to define exacerbation

| Appendix Table 2. Asthma Medications Used for Defining the Asthma Cohort |
| --- |
| 12 HR Albuterol 4 MG Extended Release Oral Tablet |
| 12 HR Albuterol 8 MG Extended Release Oral Tablet |
| 12 HR zileuton 600 MG Extended Release Oral Tablet |
| 120 ACTUAT Albuterol 0.1 MG/ACTUAT / Ipratropium Bromide 0.02 MG/ACTUAT Metered Dose Inhaler |
| 120 ACTUAT Albuterol 0.1 MG/ACTUAT / Ipratropium Bromide 0.02 MG/ACTUAT Metered Dose Inhaler [Combivent] |
| 200 ACTUAT Albuterol 0.09 MG/ACTUAT / Ipratropium Bromide 0.018 MG/ACTUAT Metered Dose Inhaler |
| 200 ACTUAT Albuterol 0.09 MG/ACTUAT Dry Powder Inhaler |
| 200 ACTUAT Albuterol 0.09 MG/ACTUAT Dry Powder Inhaler [ProAir] |
| 200 ACTUAT Albuterol 0.09 MG/ACTUAT Metered Dose Inhaler |
| 200 ACTUAT Albuterol 0.09 MG/ACTUAT Metered Dose Inhaler [ProAir] |
| 200 ACTUAT Albuterol 0.09 MG/ACTUAT Metered Dose Inhaler [Proventil] |
| 200 ACTUAT Ipratropium Bromide 0.017 MG/ACTUAT Metered Dose Inhaler |
| 200 ACTUAT Ipratropium Bromide 0.017 MG/ACTUAT Metered Dose Inhaler [Atrovent] |
| 200 ACTUAT Ipratropium Bromide 0.018 MG/ACTUAT Metered Dose Inhaler |
| 200 ACTUAT Levalbuterol 0.045 MG/ACTUAT Metered Dose Inhaler |
| 200 ACTUAT Levalbuterol 0.045 MG/ACTUAT Metered Dose Inhaler [Xopenex] |
| 400 ACTUAT Pirbuterol acetate 0.2 MG/ACTUAT Metered Dose Inhaler |
| 60 ACTUAT Albuterol 0.09 MG/ACTUAT Metered Dose Inhaler [Ventolin] |
| Accuneb Inhalant Product |
| Albuterol / Ipratropium |
| Albuterol / Ipratropium Inhalant Solution |
| Albuterol / Ipratropium Inhalant Solution [DuoNeb] |
| Albuterol 0.21 MG/ML Inhalant Solution |
| Albuterol 0.4 MG/ACTUAT Inhalant Powder |
| Albuterol 0.4 MG/ML Oral Solution |
| Albuterol 0.417 MG/ML Inhalant Solution |
| Albuterol 0.83 MG/ML Inhalant Solution |
| Albuterol 0.833 MG/ML / Ipratropium Bromide 0.167 MG/ML Inhalant Solution |
| Albuterol 1 MG/ML Inhalant Solution |
| Albuterol 2 MG Oral Tablet |
| Albuterol 4 MG Oral Tablet |
| Albuterol Inhalant Solution |
| Albuterol Oral Tablet |
| Cromolyn Inhalant Powder |
| Cromolyn Sodium 10 MG/ML Inhalant Solution |
| DuoNeb Inhalant Product |
| Ipratropium Bromide 0.2 MG/ML Inhalant Solution |
| Ipratropium Inhalant Solution |
| Levalbuterol 0.103 MG/ML Inhalant Solution |
| Levalbuterol 0.103 MG/ML Inhalant Solution [Xopenex] |
| Levalbuterol 0.21 MG/ML Inhalant Solution |
| Levalbuterol 0.21 MG/ML Inhalant Solution [Xopenex] |
| Levalbuterol 0.417 MG/ML Inhalant Solution |
| Levalbuterol 0.417 MG/ML Inhalant Solution [Xopenex] |
| Levalbuterol 2.5 MG/ML Inhalant Solution |
| Levalbuterol Inhalant Solution |
| ProAir |
| ProAir Inhalant Product |
| Proventil Inhalant Product |
| Ventolin Inhalant Product |
| Xopenex Inhalant Product |
| arformoterol 0.0075 MG/ML Inhalant Solution |
| levalbuterol tartrate |
| 120 ACTUAT Beclomethasone Dipropionate 0.04 MG/ACTUAT Metered Dose Inhaler [Qvar] |
| 120 ACTUAT Beclomethasone Dipropionate 0.08 MG/ACTUAT Metered Dose Inhaler [Qvar] |
| 120 ACTUAT Budesonide 0.08 MG/ACTUAT / formoterol fumarate 0.0045 MG/ACTUAT Metered Dose Inhaler [Symbicort] |
| 120 ACTUAT Budesonide 0.16 MG/ACTUAT / formoterol fumarate 0.0045 MG/ACTUAT Metered Dose Inhaler [Symbicort] |
| 120 ACTUAT Budesonide 0.18 MG/ACTUAT Dry Powder Inhaler [Pulmicort] |
| 120 ACTUAT Fluticasone propionate 0.044 MG/ACTUAT Metered Dose Inhaler |
| 120 ACTUAT Fluticasone propionate 0.044 MG/ACTUAT Metered Dose Inhaler [Flovent] |
| 120 ACTUAT Fluticasone propionate 0.11 MG/ACTUAT Metered Dose Inhaler |
| 120 ACTUAT Fluticasone propionate 0.11 MG/ACTUAT Metered Dose Inhaler [Flovent] |
| 120 ACTUAT Fluticasone propionate 0.22 MG/ACTUAT Metered Dose Inhaler |
| 120 ACTUAT Fluticasone propionate 0.22 MG/ACTUAT Metered Dose Inhaler [Flovent] |
| 120 ACTUAT Fluticasone propionate 0.23 MG/ACTUAT / salmeterol 0.021 MG/ACTUAT Metered Dose Inhaler [Advair] |
| 120 ACTUAT flunisolide 0.08 MG/ACTUAT Metered Dose Inhaler [Aerospan] |
| 120 ACTUAT formoterol fumarate 0.005 MG/ACTUAT / mometasone furoate 0.1 MG/ACTUAT Metered Dose Inhaler |
| 120 ACTUAT formoterol fumarate 0.005 MG/ACTUAT / mometasone furoate 0.1 MG/ACTUAT Metered Dose Inhaler [Dulera] |
| 120 ACTUAT formoterol fumarate 0.005 MG/ACTUAT / mometasone furoate 0.2 MG/ACTUAT Metered Dose Inhaler |
| 120 ACTUAT formoterol fumarate 0.005 MG/ACTUAT / mometasone furoate 0.2 MG/ACTUAT Metered Dose Inhaler [Dulera] |
| 120 ACTUAT mometasone furoate 0.1 MG/ACTUAT Metered Dose Inhaler [Asmanex] |
| 120 ACTUAT mometasone furoate 0.2 MG/ACTUAT Metered Dose Inhaler [Asmanex] |
| 120 ACTUAT mometasone furoate 0.22 MG/ACTUAT Dry Powder Inhaler |
| 120 ACTUAT mometasone furoate 0.22 MG/ACTUAT Dry Powder Inhaler [Asmanex] |
| 14 ACTUAT Fluticasone propionate 0.1 MG/ACTUAT / salmeterol 0.05 MG/ACTUAT Dry Powder Inhaler [Advair] |
| 14 ACTUAT Fluticasone propionate 0.25 MG/ACTUAT / salmeterol 0.05 MG/ACTUAT Dry Powder Inhaler [Advair] |
| 14 ACTUAT Fluticasone propionate 0.5 MG/ACTUAT / salmeterol 0.05 MG/ACTUAT Dry Powder Inhaler [Advair] |
| 14 ACTUAT fluticasone furoate 0.1 MG/ACTUAT / vilanterol 0.025 MG/ACTUAT Dry Powder Inhaler [Breo] |
| 14 ACTUAT fluticasone furoate 0.2 MG/ACTUAT / vilanterol 0.025 MG/ACTUAT Dry Powder Inhaler [Breo] |
| 14 ACTUAT mometasone furoate 0.22 MG/ACTUAT Dry Powder Inhaler |
| 28 ACTUAT salmeterol 0.05 MG/ACTUAT Dry Powder Inhaler |
| 28 ACTUAT salmeterol 0.05 MG/ACTUAT Dry Powder Inhaler [Serevent] |
| 30 ACTUAT fluticasone furoate 0.05 MG/ACTUAT Dry Powder Inhaler |
| 30 ACTUAT mometasone furoate 0.11 MG/ACTUAT Dry Powder Inhaler |
| 30 ACTUAT mometasone furoate 0.11 MG/ACTUAT Dry Powder Inhaler [Asmanex] |
| 30 ACTUAT mometasone furoate 0.22 MG/ACTUAT Dry Powder Inhaler |
| 30 ACTUAT mometasone furoate 0.22 MG/ACTUAT Dry Powder Inhaler [Asmanex] |
| 30 ACTUAT umeclidinium 0.0625 MG/ACTUAT Dry Powder Inhaler |
| 60 ACTUAT Budesonide 0.09 MG/ACTUAT Dry Powder Inhaler |
| 60 ACTUAT Budesonide 0.09 MG/ACTUAT Dry Powder Inhaler [Pulmicort] |
| 60 ACTUAT Fluticasone propionate 0.05 MG/ACTUAT Dry Powder Inhaler [Flovent] |
| 60 ACTUAT Fluticasone propionate 0.113 MG/ACTUAT / Salmeterol xinafoate 0.014 MG/ACTUAT Dry Powder Inhaler |
| 60 ACTUAT Fluticasone propionate 0.232 MG/ACTUAT / Salmeterol xinafoate 0.014 MG/ACTUAT Dry Powder Inhaler |
| 60 ACTUAT Fluticasone propionate 0.25 MG/ACTUAT Dry Powder Inhaler |
| 60 ACTUAT ciclesonide 0.08 MG/ACTUAT Metered Dose Inhaler |
| 60 ACTUAT ciclesonide 0.08 MG/ACTUAT Metered Dose Inhaler [Alvesco] |
| 60 ACTUAT ciclesonide 0.16 MG/ACTUAT Metered Dose Inhaler |
| 60 ACTUAT ciclesonide 0.16 MG/ACTUAT Metered Dose Inhaler [Alvesco] |
| 60 ACTUAT mometasone furoate 0.22 MG/ACTUAT Dry Powder Inhaler |
| 60 ACTUAT mometasone furoate 0.22 MG/ACTUAT Dry Powder Inhaler [Asmanex] |
| 60 ACTUAT tiotropium 0.00125 MG/ACTUAT Metered Dose Inhaler |
| 60 ACTUAT tiotropium 0.00125 MG/ACTUAT Metered Dose Inhaler [Spiriva] |
| 60 ACTUAT tiotropium 0.0025 MG/ACTUAT Metered Dose Inhaler [Spiriva] |
| 7 ACTUAT mometasone furoate 0.11 MG/ACTUAT Dry Powder Inhaler |
| 7 ACTUAT umeclidinium 0.0625 MG/ACTUAT / vilanterol 0.025 MG/ACTUAT Dry Powder Inhaler |
| Advair Inhalant Product |
| Aerospan Inhalant Product |
| Alvesco Inhalant Product |
| Asmanex Inhalant Product |
| Atrovent Inhalant Product |
| Breo Inhalant Product |
| Budesonide / formoterol Inhalant Powder |
| Budesonide 0.08 MG/ACTUAT / formoterol 0.0045 MG/ACTUAT Inhalant Powder |
| Budesonide 0.125 MG/ML Inhalant Solution |
| Budesonide 0.125 MG/ML Inhalant Solution [Pulmicort] |
| Budesonide 0.16 MG/ACTUAT / formoterol 0.0045 MG/ACTUAT Inhalant Powder |
| Budesonide 0.2 MG/ACTUAT Inhalant Powder |
| Budesonide 0.25 MG/ML Inhalant Solution |
| Budesonide 0.25 MG/ML Inhalant Solution [Pulmicort] |
| Budesonide 0.5 MG/ML Inhalant Solution |
| Budesonide 0.5 MG/ML Inhalant Solution [Pulmicort] |
| Budesonide Inhalant Powder |
| Combivent Inhalant Product |
| Dulera Inhalant Product |
| Flovent Inhalant Product |
| Mometasone Dry Powder Inhaler |
| Mometasone Inhalant Product |
| Pulmicort Inhalant Product |
| Qvar Inhalant Product |
| Serevent Inhalant Product |
| Symbicort Inhalant Product |
| Theophylline 200 MG Extended Release Oral Tablet |
| Theophylline 300 MG Extended Release Oral Tablet |
| Theophylline 5.33 MG/ML Oral Solution |
| Xolair |
| fluticasone Inhalant Solution |
| formoterol fumarate 0.01 MG/ML Inhalant Solution |
| formoterol fumarate 0.012 MG Inhalant Powder |
| mepolizumab 100 MG Injection |
| omalizumab |
| omalizumab 150 MG Injection |
| omalizumab 150 MG Injection [Xolair] |
| tiotropium 0.018 MG Inhalant Powder |
| tiotropium 0.018 MG Inhalant Powder [Spiriva] |
| Singulair Oral Product |
| montelukast 10 MG Oral Tablet |
| montelukast 10 MG Oral Tablet [Singulair] |
| montelukast 4 MG Chewable Tablet |
| montelukast 4 MG Chewable Tablet [Singulair] |
| montelukast 4 MG Oral Granules |
| montelukast 4 MG Oral Granules [Singulair] |
| montelukast 5 MG Chewable Tablet |
| montelukast 5 MG Chewable Tablet [Singulair] |
| montelukast Oral Tablet |
| zafirlukast 10 MG Oral Tablet |
| zafirlukast 20 MG Oral Tablet |

| Appendix Table 3. Comparison Among Different Models | | | | | | | | |
| --- | --- | --- | --- | --- | --- | --- | --- | --- |
|  | Unadjusted | | Adjusted for SES Score | | Adjusted for Race/Insurance/  Sex/Age | | Adjusted for Number of Encounter /Medication/  Obesity/Atopy | |
|  | HR | 95% CI | HR | 95% CI | HR | 95% CI | HR | 95% CI |
| AG Models | 0.98 | 0.94, 1.01 | 0.98 | 0.94, 1.02 | 0.99 | 0.95, 1.02 | 0.99 | 0.96, 1.02 |
| PWP Models | 0.99 | 0.97, 1.01 | 0.99 | 0.96, 1.01 | 0.99 | 0.97, 1.01 | 0.99 | 0.96, 1.01 |
| Frailty Models | 0.98 | 0.94, 1.02 | 0.98 | 0.94, 1.02 | 0.98 | 0.95, 1.02 | 0.98 | 0.95, 1.02 |
| AG: Andersen-Gill model PWP: Prentice, Williams and Peterson model  HR: Hazard ratio 95% CI: 95% Confidence Interval SES: Socio-economic Status | | | | | | | | |
